# Surfaces of Atmospheric Droplet Models Probed with Synchrotron XPS on a Liquid Microjet

**DOI:** 10.1021/acs.accounts.3c00201

**Published:** 2023-12-29

**Authors:** Nønne L. Prisle

**Affiliations:** Center for Atmospheric Research, University of Oulu, P.O. Box 4500, Oulu 90014, Finland

## Abstract

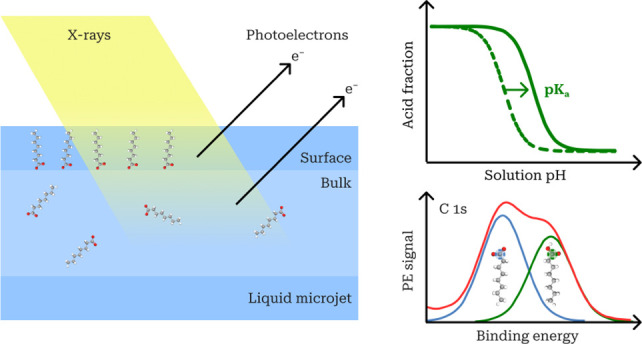

The atmosphere is a key part of the earth system comprising myriad chemical species in all basic forms of matter. Ubiquitous nano- and microscopic aerosol particles and cloud droplets suspended in the air play crucial roles in earth’s climate and the formation of air pollution. Surfaces are a prominent part of aerosols and droplets, due to the high surface area to bulk volume ratios, but very little is known about their specific properties. Many atmospheric compounds are surface-active, leading to enhanced surface concentrations in aqueous solutions. Their distribution between the surface and bulk may determine heterogeneous chemistry and many other properties of aerosol and cloud droplets, but has not been directly observed.

We used X-ray photoelectron spectroscopy (XPS) to obtain direct molecular-level information on the surface composition and structure of aqueous solutions of surface-active organics as model systems for atmospheric aerosol and cloud droplets. XPS is a vacuum-based technique enabled for volatile aqueous organic samples by the application of a high-speed liquid microjet. In combination with brilliant synchrotron X-rays, the chemical specificity of XPS allows distinction between elements in different chemical states and positions within molecular structures. We used core-level C 1s and N 1s signals to identify the alkyl and hydrophilic groups of atmospheric carboxylic acids, alkyl-amines, and their conjugate acids and bases. From this, we infer changes in the orientation of surface-adsorbed species and quantify their relative abundances in the surface. XPS-derived surface enrichments of the organics follow trends expected from their surface activities and we observed a preferential orientation at the surface with the hydrophobic alkyl chains pointing increasingly outward from the solution at higher concentrations. This provides a first direct experimental observation of well-established concepts of surface adsorption and confirms the soundness of the method.

We mapped relative abundances of conjugate acid−base pairs in the aqueous solution surfaces from the respective intensities of distinctive XPS signals. For each pair, the protonation equilibrium was significantly shifted toward the neutral form in the surface, compared to the bulk solution, across the full pH range. This represents an apparent shift of the p*K*_a_ in the surface, which may be toward either higher or lower pH, depending on whether the acid or base form of the pair is the neutral species. The surface shifts are broadly consistent with the relative differences in surface enrichment of the individual acid and base conjugates in binary aqueous solutions, with additional contributions from nonideal interactions in the surface. In aqueous mixtures of surface-active carboxylate anions with ammonium salts at near-neutral pH, we found that the conjugate carboxylic acids were further strongly enhanced. This occurs as the coadsorption of weakly basic carboxylate anions and weakly acidic ammonium cations forms ion-pair surface layers with strongly enhanced local abundances, increasing the probability of net proton transfer according to Le Chatelier’s principle. The effect is stronger when the evaporation of ammonia from the surface further contributes to irreversibly perturb the protonation equilibrium, leaving a surplus of carboxylic acid. These surface-specific effects may profoundly influence atmospheric chemistry mediated by aqueous aerosols and cloud droplets but are currently not taken into account in atmospheric models.

## Key References

- PrisleN. L.; OttossonN.; ÖhrwallG.; SöderströmJ.; MasoM. D.; BjörneholmO.Surface/bulk partitioning and acid/base speciation of aqueous decanoate: direct observations and atmospheric implications. Atmos. Chem. Phys.2012, 12, 12227–12242.10.5194/acp-12-12227-2012(1)*Surface-sensitive X-ray photoelectron spectroscopy in combination with synchrotron radiation showed that the formation of a decanoate–ammonium ion-pair surface layer with enhanced local concentrations leads to a strong enhancement of decanoic acid from proton transfer in accordance with Le Chatelier’s principle.*
- ÖhrwallG.; PrisleN. L.; OttossonN.; WernerJ.; EkholmV.; WalzM.-M.; BjörneholmO.Acid–Base Speciation of Carboxylate Ions in the Surface Region of Aqueous Solutions in the Presence of Ammonium and Aminium Ions. J. Phys. Chem. B2015, 119, 4033–4040.10.1021/jp509945g25700136 (2)*Surface-specific carboxylic acid enhancement was observed for different mixtures of carboxylate anions with ammonium or alkyl-ammonium cations and depends on the surface activity (alkyl chain length) of the carboxylate anion with an additional contribution from the possible evaporation of the corresponding amine.*
- WernerJ.; PerssonI.; BjörneholmO.; KaweckiD.; SaakC.-M.; WalzM.-M.; EkholmV.; UngerI.; ValtlC.; CalemanC.; ÖhrwallG.; PrisleN. L.Shifted equilibria of organic acids and bases in the aqueous surface region. Phys. Chem. Chem. Phys.2018, 20, 23281–23293.10.1039/C8CP01898G30191936 PMC6146375(3)*The protonation equilibria of carboxylic acid and alkyl-amine conjugate acid−base pairs are significantly shifted toward the neutral form in the aqueous solution surface across the full bulk solution titration curves and consistent with relative differences in individual surface activities.*

## 1 Aerosols and Cloud Droplets in the Atmosphere

The atmosphere is a key part of the earth system. It contains the air we breathe, regulates weather and climate, and supports critical infrastructure. Earth’s atmosphere comprises myriad chemical species in all basic forms of matter. Water is found as vapor, cloud and fog droplets, snow, or ice crystals. Condensed atmospheric phases are present as ubiquitous nano- and microscopic aerosol particles and droplets suspended in the air. These aerosols originate from numerous natural and anthropogenic processes and may comprise a wide range of both organic and inorganic chemical species.^4,5^

Interactions with water govern many effects of aerosols in the atmosphere. Depending on ambient conditions and aerosol composition and phase state, water can condense onto the aerosol surfaces, forming droplet solutions, which grow into large cloud droplets that eventually precipitate.^6,7^ Every cloud droplet in the atmosphere contains within it the aerosol seed from which it formed. Aerosols and clouds play crucial roles in earth’s climate, but the underlying mechanisms remain the most poorly constrained, severely affecting projections of future climate change.^8^ Aerosols and cloud droplets provide media for condensed-phase and heterogeneous chemistry throughout the atmosphere, with significant contributions to the evolution of atmospheric composition.^9^ Air pollution in particular causes millions of premature deaths and estimated welfare losses of trillions of euros every year.^10^ A detailed understanding of the atmosphere and its key constituents and processes is vital for robust projections to support the mitigation of both air pollution and climate change.

### 1.1 Role of Droplet Surfaces

**Figure 1:**
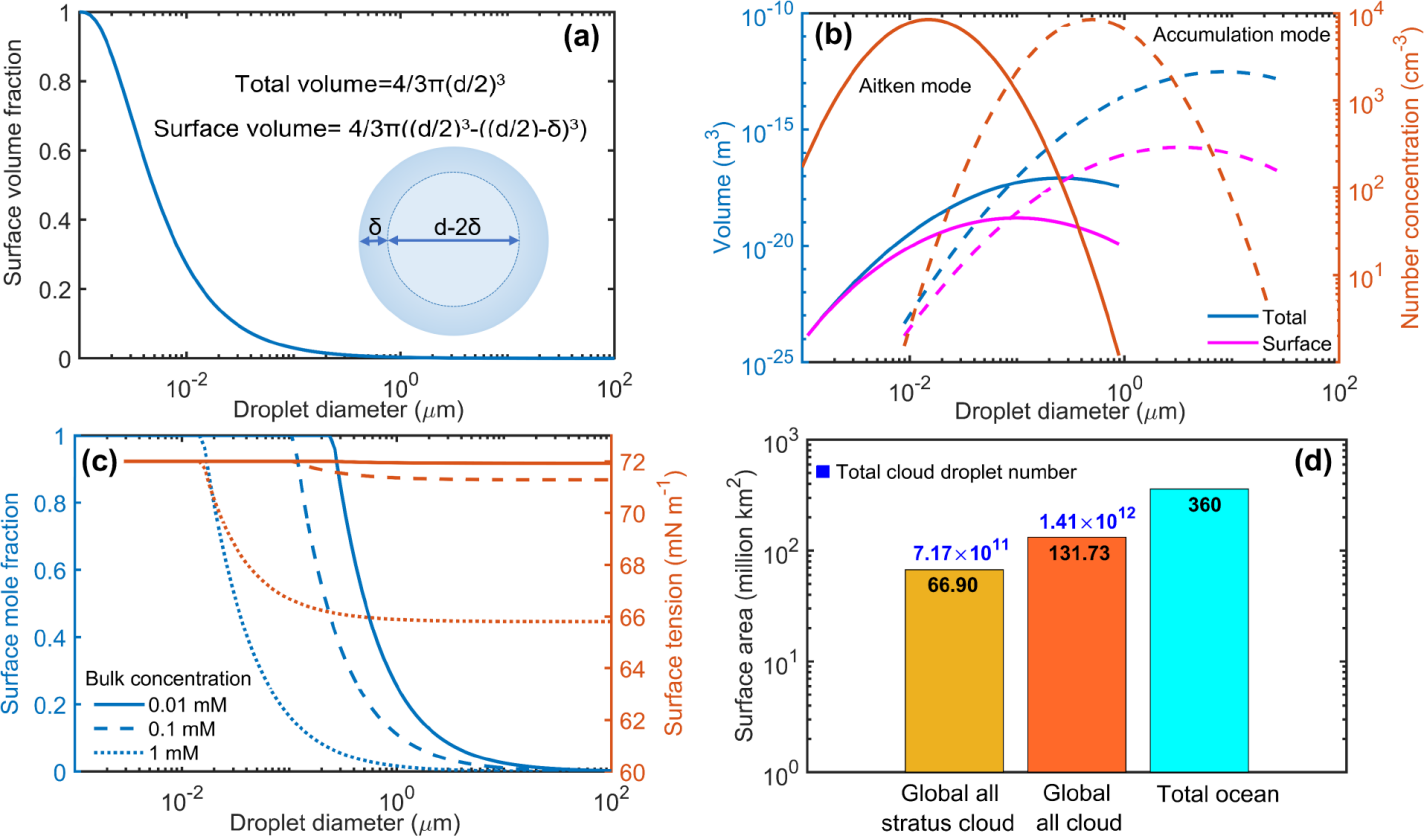
Importance of droplet surfaces. (a) Variation of the surface volume fraction with droplet diameter, assuming a surface thickness of δ = 1 nm. (b) Total and surface volume (left axis) calculated for common atmospheric aerosol populations from refs (15) and (16) (right axis). (c) Fraction of surface-adsorbed solute (left axis), assuming a surface of thickness δ = 1 nm with enrichment factor *E*^σ^ = 100, compared to the solution bulk, and surface tension resulting from concurrent bulk depletion (right axis), calculated using parametrizations from refs (11) and (17). (d) Total surface area of clouds, calculated with number concentrations for stratus and all clouds from ref (18) and a fixed droplet distribution from ref (19) and, for comparison, of earth’s oceans, calculated with data from ref (20).

Surfaces are a prominent part of atmospheric aerosols due to their high surface area (*A*) to bulk volume (*V*) ratios. The total surface area of cloud droplets in the atmosphere may be similar to that of all of the oceans (Figure 1d). In nano- and microscopic phases, both the surface and bulk are finite and *A*/*V* may be orders of magnitude greater than for macroscopic systems.^7,11−13^ With a finite thickness (δ), for example given by the dimensions of a molecular monolayer,^1,14^ the surface constitutes an increasing fraction of the total condensed-phase volume as dimensions decrease in the submicrometer range (Figure 1a). For aerosol populations similar to those observed in the atmosphere,^15,16^ a significant fraction of the total volume can therefore be comprised by their surfaces (Figure 1b).

Many atmospheric compounds, in particular amphiphilic organics, are surface-active in aqueous solutions, including aerosol and cloud droplets.^6,13,21−23^ Surface-active components preferentially adsorb at the surface, resulting in enhanced surface concentrations (activity) with respect to the interior (bulk) and a concentration gradient between the surface and bulk phases of a solution. Organic compounds often have the ability to lower the surface tension of an aqueous solution, compared to that of pure water. With enhanced surface activity, such compounds may reduce the surface tension even more efficiently than if homogeneously mixed in the solution. Inorganic ions can also display nonisotropic distributions at the aqueous surface,^24,25^ but their surface propensities are often not well-constrained in atmospheric mixtures.

Nano- and microscopic droplets are mesoscale bulk solutions, where strong mutual impacts of surface/bulk equilibria must be taken explicitly into account.^13^ For example, enhanced surface activity can significantly deplete the finite amount of solute from the bulk phase.^11,13,17^ The resulting distribution of substance between the surface and bulk phases is referred to as surface/bulk *partitioning*.^6,7^ The composition of both phases and composition-dependent solution properties, including surface tension, density, conductivity, and chemical reactivity, may significantly change with surface/bulk partitioning.^7,13,26,27^ Due to the strong variation of *A*/*V* with size, surface/bulk partitioning and composition-dependent properties will depend on the dimensions of nano- and microscopic solutions (Figure 1c). Bzdek et al.^12^ presented the first direct measurements of size-dependent surface tensions for picoliter aqueous surfactant droplets suspended in air, which were significantly higher than those of macroscopic solutions with identical total compositions.

Both the surface tension and amount of solute in the bulk affect the potential of aqueous droplets to grow by water condensation into cloud droplets.^7,13^ Two opposing mechanisms together define a threshold droplet size and ambient water saturation, which must be exceeded to *activate* the droplet for diffusion-limited growth.^6^ The finite curvature radii of microscopic solutions lead to elevated pressures (Kelvin effect), which scale with the surface tension. Dissolved solutes reduce the water vapor pressure (Raoult effect). Both of these mechanisms can be dramatically impacted by size-dependent surface/bulk partitioning.^6,7,11,13,17,26−29^ A significant climate effect has been estimated from altered cloud properties by surface-active droplet components^21,30,31^ but is not well-constrained. A major reason is insufficient knowledge on the variations of the droplet state and surface tension with environmental conditions.

The surface activity of atmospheric aerosol components may also have important implications for aqueous chemistry occurring as droplets grow and shrink during multiple cycles of cloud processing. Similarly as for water vapor, the Kelvin effect and surface tension will influence the condensation–evaporation of other volatile atmospheric species across the droplet surface. This will affect the formation and composition of so-called secondary aerosol mass, which remains a major source of uncertainty in assessing overall atmospheric aerosol and cloud effects.^32^ The surface tension of cloud droplets may also impact rates of pressure-sensitive chemical reactions in the droplet phase.^13,33,34^ Surface/bulk partitioning can dramatically alter rate-determining concentrations in both bulk and surface phases and may thereby directly impact chemical reactions in submicrometer droplets.^1,13,35^ Heterogeneous chemistry on the surfaces of aerosol and cloud droplets, with unique reaction pathways, rates, and products, compared to gas and condensed bulk-phase reactions, is increasingly recognized as potentially significant in the global atmospheric budget.^36,37^ However, such reactions are currently not well-constrained and rarely explicitly accounted for in atmospheric models.

### 1.2 Atmospheric Aerosol and Cloud Droplet Models

Very little is known about the specific properties of atmospheric surfaces. Information on the distinct surface composition is obscured in aerosol samples characterized as bulk aggregates^21^ or with bulk-sensitive methods, such as aerosol mass spectrometry.^38^ Quantitative surface-sensitive methods generally require macroscopic samples and experimental conditions, such as high vacuum, which do not readily accommodate volatile aerosol and cloud droplet constituents.^1,39,40^

Complex atmospheric aerosol mixtures introduce exponentially growing composition-dependent parameter spaces. Process-level studies therefore typically focus on simple model mixtures of a single, or few, key atmospheric species with well-defined and stable sample properties. Even when compositions or suitable proxies are identified, direct characterization of size-dependent composition–property relations is extremely challenging.^12,41^ Models are therefore needed to connect bulk measurements to surface compositions and macroscopic measurements to microscopic properties.^13,14,17^

Major classes of atmospheric organics comprise carboxylic acids, amines, organosulfates, and their salts, and alcohols. The most common inorganic ions are Na^+^, NH_4_^+^, Cl^–^, and SO_4_^2–^. Since liquid water is an integral part of atmospheric aerosols and cloud droplets, it is important to establish the properties of both organic and inorganic components and their mixtures in aqueous solutions. Concentrations of individual compounds vary by many orders of magnitude under ambient conditions.^7,13,26,27^ Organic compounds, in particular, may be highly dilute due to limited aqueous solubilities and the formation of many different species in atmospheric chemical reactions.

Molecular dynamics (MD) simulations allow a molecular-level investigation of the surface adsorption of solutes and the structure of the surface region.^3,42^ However, the system size required to represent dilute aqueous solutions and unknown interaction parameters pose challenges for atmospheric mixtures. Concentration gradients between the bulk and surface are represented by a very small number of solute entities, with related uncertainty. The *A*/*V* of systems treated in MD simulations may also not represent either atmospheric droplets or macroscopic solutions to validate concentration-dependent properties.^12,13,26,27,43^

Several thermodynamic surface/bulk partitioning models have been used to evaluate cloud droplet formation by surface-active aerosols.^14,26,27^ A few are predictive, which is necessary to describe droplet states not accessed in experiments. These models are often based on Gibbs thermodynamics, where surface adsorption is described as an excess with respect to an idealized two-dimensional surface.^7,13^ The surface excess has no physical volume and does not represent the full composition or structure of the surface. Typically, only the adsorption of a single component is treated, whereas many surface-active species have been identified to coexist in atmospheric aerosol samples.^22,23^ Our Monolayer surface model^11,14,26,27^ predicts the full composition of both surface and bulk phases. In principle, the model can be constrained by independent experimental (surface tension and density) data that captures all interactions in the solution. However, such data is often not available for atmospheric aqueous solutions and is approximated with simplified equations of state.^13,14^

## 2 Surface-Sensitive XPS on a Liquid Microjet

**Figure 2:**
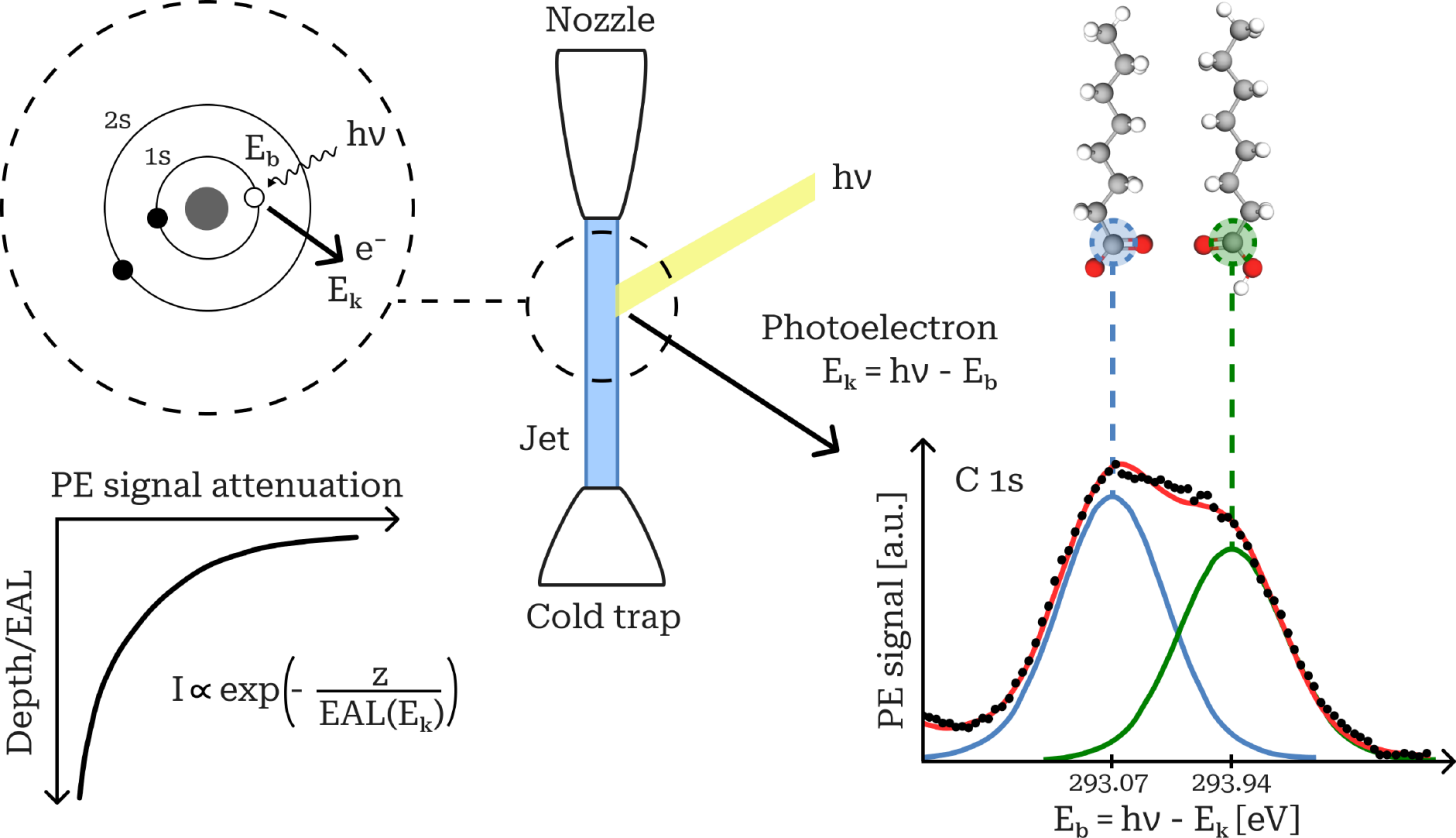
XPS on a liquid microjet. Measurement principle, example C 1s spectrum for an octanoate (left fitted peak)–octanoic acid (right fitted peak) aqueous mixture (data from ref (2)), and dependence of signal attenuation (probing depth) on EAL.

X-ray photoelectron spectroscopy (XPS) is a surface-sensitive technique capable of providing direct molecular-level information with high chemical specificity. Photoelectron spectroscopy utilizes the photoelectric effect to ionize a sample from inelastic collisions with photons. The emitted photoelectrons are characterized in terms of kinetic energy (*E*_k_), from which their binding energy (*E*_b_) within the sample is determined as the difference from the known ionizing photon energy (*hν*), *E*_b_ = *hν* – *E*_k_. By using X-ray photons, atomic-like core-level orbitals can be ionized and their electron binding energies reflect the chemical composition and local environment of the probed sample region. In combination with a tunable synchrotron light source, a wide range of chemical species and states can be accessed with high selectivity. Furthermore, the probing depth into the sample can be varied to optimize the surface sensitivity for each target species^1−3,39,44^ via the effective attenuation length (EAL), which depends on *E*_k_.^45^ The photoelectron signal intensity (*I*) originating at a given depth (*z*) below the surface decays exponentially as  (Figure 2).

Due to strong attenuation of the photoelectron signal also from gas-phase collisions, XPS is a vacuum-based technique. Both water and many other atmospheric species have finite vapor pressures and are either volatile or semivolatile under ambient conditions.^38^ By probing the sample as a high-speed liquid microjet,^46^ in combination with differential pumping of the experimental region, XPS can be applied to aqueous solutions of compounds with immediate atmospheric relevance.^1−3,42,47,48^ Minimum aqueous concentrations that can be studied depend on experimental conditions, including the surface propensity and ionization cross section of the target species and intensity of the ionizing light source. Ionization cross sections vary by several orders of magnitude among different elements, for orbitals within a given element, and with respect to photon energies.^49^ With high-brilliance synchrotron X-rays, XPS can distinguish elements in chemical states corresponding to different positions within molecular structures, oxidation or protonation states, and solvation environments (Figure 2).

### 2.1 XPS Experiments for Cloud Droplet Model Solutions

In a series of experiments, we studied the surface adsorption and speciation of surface-active atmospheric organics with Brønsted acid–base character in aqueous solution. Results for conjugate pairs of monocarboxylic acids and alkyl-amines are highlighted here. We focused on the effects of varying surface activity,^2^ chemical functionality of the hydrophilic group,^3^ and aqueous bulk solution state in terms of pH^3^ and mixing with common atmospheric inorganic salts comprising Na^+^, NH_4_^+^, Cl^–^, and SO_4_^2–^ ions.^1,2^

XPS experiments were performed at the Swedish National Synchrotron Radiation Laboratory MAX-lab, beamline I411. Aqueous sample stock solutions were prepared with varying pH and solute concentrations between a few millimolar and a few molar, representative of growing and activating cloud droplets.^6,7,11,13,17,26,27^ These concentrations were below the solubility limit or a possible critical micelle concentration (CMC) of all solutes, to ensure jet stability and avoid phase transitions which would decouple the observed properties from the concentration. The aqueous samples were introduced into the experimental region as a jet with a velocity of ∼25 m s^–1^, formed by a nozzle with a diameter of ∼20 μm. The microjet intersected with X-rays from the beamline a few millimeters downstream from the nozzle, in the region of stable laminar flow. The temperature of the microjet at the intersection point was estimated to be 5 ± 5 °C. Afterward, the sample was frozen in a liquid-nitrogen-cooled trap to minimize pressure building from evaporation.

The kinetic energies (*E*_k_) of photoelectrons emitted from ionization by the X-rays are recorded with a spectrometer. The photoelectrons enter the spectrometer through a skimmer placed a few millimeters above the surface of the microjet. This makes it possible to operate the spectrometer at much lower pressure than the experimental region. The liquid jet was perpendicular to the propagation axis of the X-rays and the spectrometer detection axis. The latter was at a 54.7° angle, also known as the ”magic angle”, relative to the polarization plane of the X-rays to minimize angular distribution effects in the recorded photoelectron signal.^50^

An XPS spectrum consists of photoelectron (PE) signal intensities recorded at a fixed X-ray energy across a well-defined range of *E*_k_, corresponding to relevant *E*_b_ for a given target core-level orbital (Figure 2). In each spectrum, the majority of photoelectrons cluster around one or more *E*_k_ values, forming spectral peaks representative of different chemical species or molecular environments. Recorded spectra for each target orbital are fitted to obtain peak positions *E*_b_ and areas (intensities) *I*, which are used to identify distinct chemical species or states and quantify their relative abundances in the probed sample region.

Carboxylic acids and their conjugate bases (carboxylate anions) were probed via the C 1s PE signals from the alkyl chain (−**C**H_2_−) and the carboxylic acid group (−**C**OOH) and its deprotonated carboxylate form (−**C**OO^–^). Alkyl amines and their conjugate acids were observed via both C 1s and N 1s signals from the alkyl chain (−**C**H_2_−) and the amine (−**N**H_2_) and ammonium (−**N**H_3_^+^) groups, respectively. Binding energies are calibrated with a well-known reference, such as the liquid water 1b_1_ peak at 11.16 eV.^51^ XPS allows a clear distinction between the conjugates of each acid−base pair from the chemical shifts of *E*_b_ peaks in the recorded spectra (Figure 2).

For a given *E*_k_, PE signal intensities *I* are directly proportional to the abundances (*c*) of ionized chemical species or states in the probed sample region. Additionally, PE intensities are affected by the photoionization cross section of the target orbital, the density profile ρ(*z*) of the species with respect to the surface (*z* = 0), and the depth of the probed region determined by the photoelectron EAL(*E*_k_).^44,52^ To the extent that these factors can be accounted for, PE intensities can be compared between different samples when measured during unchanged conditions. However, in practice this is nontrivial. We monitor the stability of the measurements, including sample injection, synchrotron radiation, and alignment of the liquid jet, X-ray beam, and skimmer, across the acquisition time via an internal reference. The EAL is not exactly known for aqueous solutions, and quantitative comparison of spectra for very different *E*_k_ values has significant uncertainty. The recorded PE signals originate from both the surface and bulk (near-surface) regions in the sample. By using photon energies to yield C 1s and N 1s photoelectrons with *E*_k_ ≈ 70 eV and EAL ≈ 10 Å, the relative contribution from species in the surface to the recorded PE intensities is close to being maximized.^53^

Absolute surface abundances (*c*^σ^) are estimated in terms of surface enrichment factors (*E*^σ^ = *c*^σ^/*c*^aq^) for each species with respect to their known bulk concentrations (*c*^aq^). We impose a simple two-layer model of the surface,^1,3,42^ resolving observed PE signals into contributions from the surface (*I*^σ^) and bulk (*I*^aq^)
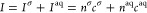
1where *n*^σ^ and *n*^aq^ represent the sensitivity of measurements to the surface and bulk layers, respectively. Surface enrichments are then obtained from^1,3^
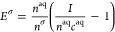
2With given EAL and surface thickness δ, *I*^σ^ and *I*^aq^ are estimated^3^ for an isotropic species, where *c*^σ^ = *c*^aq^, to determine the relative sensitivity as *n*^σ^/*n*^aq^ = *I*_isotropic_^σ^/*I*_isotropic_^aq^ via eq 1. δ is obtained from MD density profiles^3,42^ or approximated with a molecular monolayer.^1,11,14^ For highly surface sensitive measurements, the two-layer model typically yields . By comparing to PE signals from similar chemical states in species with little or no surface activity (surface depleted), where *c*^σ^ = 0, eq 1 further yields *n*^aq^ = *I*_depleted_/*c*_depleted_^aq^. For example, we have compared carboxylic C 1s intensities from surface-active carboxylate anions to those of formate.

Although peak shapes and absolute chemical shifts may be affected by solvent effects^54^ for both neutral and ionic solutes, these were not explicitly investigated. Observed *E*_b_ values agree well with previous references, and conjugates are readily identified in each spectrum from relative shifts of their corresponding peaks. PE intensities are obtained as integral areas of fitted spectral peaks, which are assumed to be unaffected by peak shape due to the proportionality of signal and abundance.

## 3 Key Results

### 3.1 Direct Observations of Surface Adsorption

**Figure 3:**
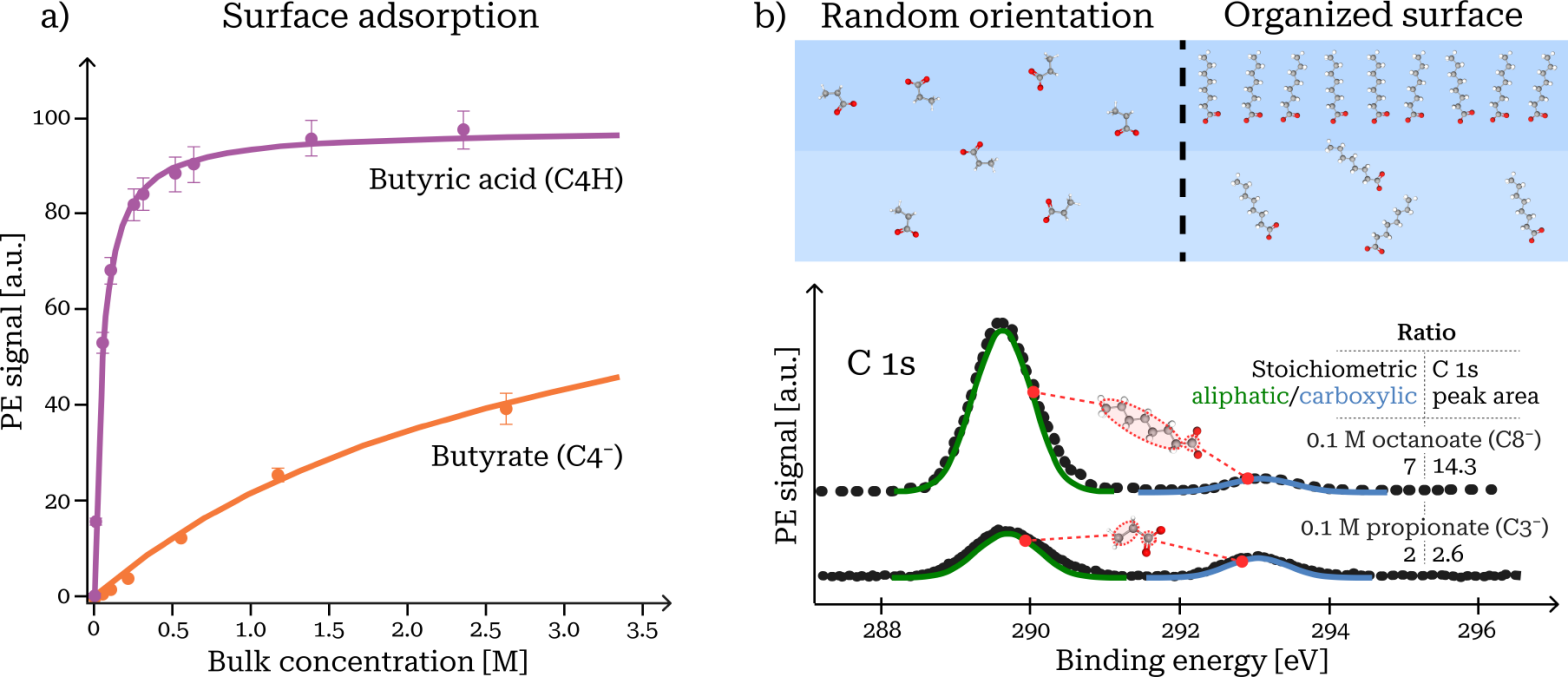
Direct observation of surface adsorption. (a) Langmuir-like adsorption curves in terms of surface abundances from XPS spectra for bytyric acid and butyrate anions (data from ref (3)). (b) Orientation with respect to the surface observed for octanoate and propionate anions via relative alkyl/carboxylic group C 1s peak areas (data from ref (2)).

We observed clear PE signals from our samples, confirming sufficient surface concentrations and photoionization cross sections of the target species.^1−3^ XPS spectra of surface-active atmospheric organics showed significant enrichment in the aqueous surface, in accordance with expectations from adsorption theory.^7,11,14,17,26,27,42,55^ The two-layer model yielded surface enrichment factors between a few multiples and several orders of magnitude for the investigated monocarboxylic acids, alkyl-amines, and their conjugates.^1−3^ Greater *E*^σ^ values were observed for species expected to be more surface-active in aqueous solution (Figure 3a). For each conjugate pair, the neutral species was more strongly enriched in the surface than the charged form. We observed stronger surface enrichments of alkyl-ammonium ions compared to carboxylate ions with similar alkyl chain lengths.^3^ XPS-derived surface abundances increased with bulk concentration in solution and eventually saturated, akin to Langmuir surface-adsorption curves.^3^ Surface saturation occurred at lower bulk concentrations and the fractional surface saturation at a given bulk concentration was higher for species expected to be more surface-active. No significant impact on organic surface adsorption, in terms of salting-out and common-ion effects from mixing with inorganic cosolutes,^56,57^ was observed for the investigated concentrations and relative mixing states.^1,2^

Surface enrichments obtained from XPS spectra depend on the nonisotropic density profiles of the surface-active species in the probed sample region and the surface sensitivity of the measurements. The surface activity of the investigated amphiphilic species stems from a sensitive balance between hydrophobic interactions of the alkyl chain and hydrophilic interactions of the polar or charged groups in the solution.^3^ A higher relative permittivity is required to stabilize highly polar or charged species than less polar molecular species. The relative permittivity is likely lower in the outermost surface region than in the bulk aqueous solution due to the enrichment of less polar organic species and reduced overall density. This explains the relatively higher propensity of molecular conjugates for the immediate surface region. Charged species can be stabilized in the surface region through the formation of ion pairs reducing the overall local charge. The local charge screening is more effective when ions are closer and could therefore be less efficient for carboxylate than alkyl-ammonium ions due to stronger aqueous-phase hydration.

Surface adsorption can be seen as full or partial separation into organic surface (σ) and aqueous bulk (aq) phases with distinct compositions.^14,28,38^ Here, the activity coefficients of each species *i*
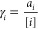
3where *a*_*i*_ and [*i*] are the corresponding activity (with respect to a given reference state) and concentration of *i*, will vary significantly.^57^ For more surface-active species, γ_*i*_ is reduced more in the surface, compared to the bulk, than for the less-surface-active species. Therefore, greater *E*^σ^ = [*i*]^σ^/[*i*]^aq^ values are necessary to yield the same activity *a*_*i*_^σ^ = *a*_*i*_^aq^ at equilibrium with a given [*i*]^aq^. Activity coefficients are expected to decrease with H-bond-forming ability and increase with the length of the alkyl chain in the dilute aqueous bulk and *vice versa* for the organic surface.^57^

For all surface-adsorbed organics, we observed a preferential orientation with the hydrophobic alkyl chains pointing away from and the hydrophilic groups embedded into the solution. This is evident as an enhancement of the alkyl/hydrophilic group PE intensity ratio, relative to the stoichiometric ratio expected for free, randomly oriented solutes. For example, stoichiometric C 1s peak ratios between the carboxylate/carboxylic and alkyl carbons would be 1:7 for octanoate and 1:2 for propionoate (Figure 3b). With preferential orientation, PE signals from alkyl and hydrophilic groups originate at different depths *z* and are attenuated to different degrees as . The alkyl signal is increasingly enhanced compared to the carboxylic signal for higher bulk concentrations, indicating that the alkyl chain is increasingly perpendicular to the surface closer to saturation. This confirms, by direct observation, the expected behavior for a surface state above the dilute (Traube’s law) limit.^58^

### 3.2 Apparent Shift in Surface Acidity

Carboxylic acids and alkyl-ammonium cations act as Brønsted acids (proton donors) in aqueous solution, and their conjugate carboxylate anions and alkyl-amines act as Brønsted bases (proton acceptors). The hydrolysis of monoprotic acid HA to form its conjugate base B is given by
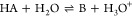
4with the acid dissociation equilibrium constant
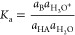
5where *a*_H_3_O^+^_, *a*_H_2_O_, *a*_B_, and *a*_HA_ are the activities of H_3_O^+^, H_2_O, B, and HA, respectively. In dilute aqueous bulk solutions, approximating *a*_H_2_O_ ≈ 1 and other activities by the corresponding concentrations yields the Henderson–Hasselbalch equation
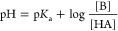
6

**Figure 4:**
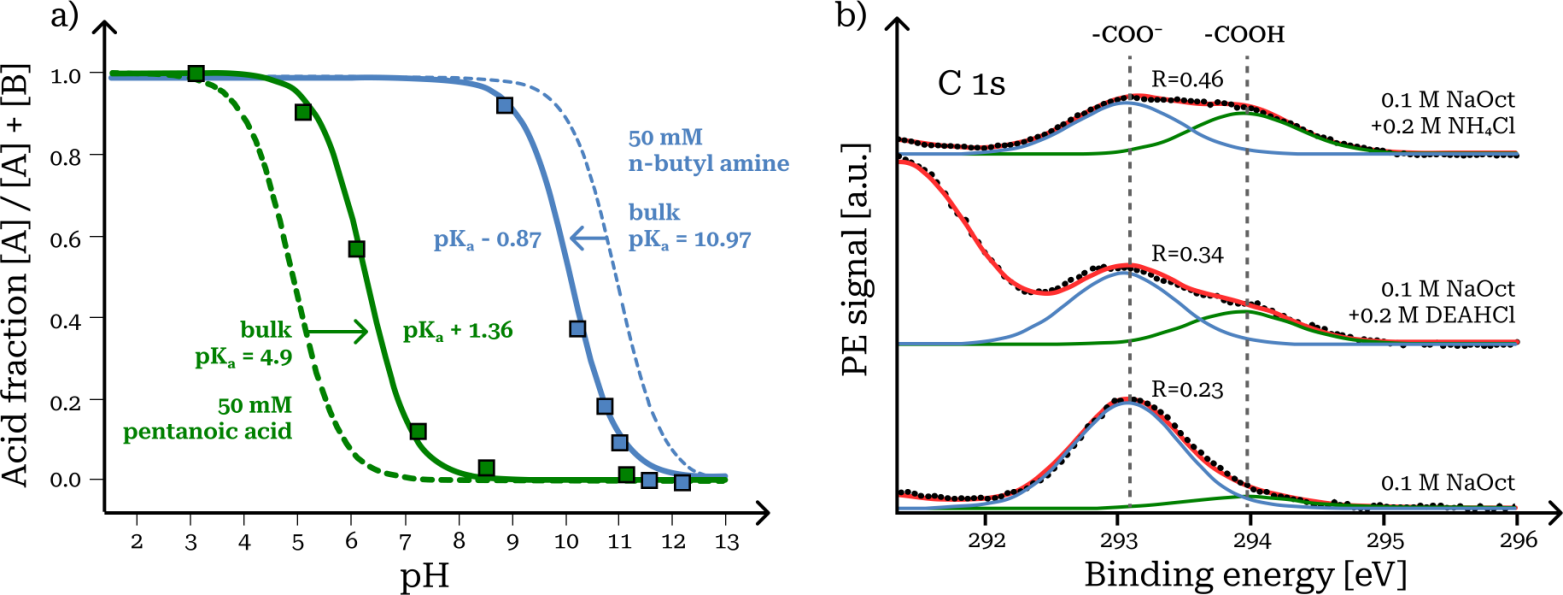
Surface-specific protonation. (a) Shifted equilibria toward the neutral species of conjugate acid−base pairs and apparent p*K*_a_ in the surface of aqueous solutions (data from ref (3)). (b) Enhanced protonation of octanoate in aqueous mixtures with ammonium and diethanolammonium cations (data from ref (2)).

We mapped the relative abundances  of conjugate acid−base pairs in the surface of dilute aqueous solutions from the respective intensities of distinctive XPS spectral peaks. For each pair, observed  values were significantly shifted toward the neutral form in the surface compared to the aqueous bulk solution at a given pH. The shifts were observed for both proton donors (butyric and pentanoic acid) and acceptors (*n*-butyl and *n*-hexyl amine) as neutral species and across a wide range of bulk pH (2–13). The relative intensities of XPS peaks corresponding to acid and base forms of each conjugate pair were fitted as a function of bulk solution pH to a reformulation of eq 6 in terms of the acid fraction  (Figure 4a). For each acid−base pair, the fitted titration curves represent an *apparent* shift of the p*K*_a_ (as the pH where ) in the surface, compared to a dilute bulk aqueous solution. The shift may be toward either higher or lower pH, depending on whether the acid or base form of the pair is the neutral species.

The magnitude of the shift varies with bulk concentration and surface activities of the conjugates. For the investigated samples, apparent p*K*_a_ shifts are roughly between 0.5 and >1 pH unit.^3^ Considerable intensity of the protonated acidic form was previously observed in XPS spectra from aqueous formic acid^59^ and sodium propionate,^2^ octanoate,^2^ and decanoate^1^ salts, at pH up to five units higher than the bulk p*K*_a_. This corresponds to a shift in  of up to roughly 4 orders of magnitude. Suppressed dissociation at the aqueous surface was also observed with XPS for highly concentrated nitric^60^ and sulfuric^54^ acid, involving similar preferential stabilization of the neutral species. Our measurements for dilute aqueous solutions suggest strong contributions from the surface activity of both organic acids and bases across the full titration curve pH range and further influence of ion pairing with cosolutes.

These observations do not *per se* imply that the surface itself is more acidic or basic than bulk water.^61^ First, we directly observe the abundances of the surface-active conjugate acid−base pairs but not water or its conjugate acid H_3_O^+^ and base OH^–^. Second, contrary to the dilute aqueous bulk, the probed surfaces are predominantly organic phases with water present as a solute.^11,17,26−28,48^ The degree of acid protonation in the aqueous surface is expected to vary due to different hydration enthalpies of protonated and deprotonated species.^62^ These thermodynamic properties will further change for a predominantly organic surface phase. The observed shifts in  and apparent p*K*_a_ in the surface can be attributed to differences in the surface propensities and/or radial density profiles of the acid and base forms. If the nonisotropic density profile ρ(*z*) of the charged form of the pair peaks deeper below the surface, then this will bias the observed PE signal to reflect a lower relative abundance in the surface than for the neutral form. However, comprehensive angle-resolved XPS experiments for environmental organosulfates in aqueous solutions demonstrated that relative surface abundances were due to differences in peak intensity (ρ), rather than peak depth (*z*), of the density profiles with respect to the surface.^63^

Given that the observed  values indeed reflect the relative composition of the surface, the apparent shifts in p*K*_a_ can be rationalized in terms of the relative change in activity coefficients of the conjugates between the organic surface and aqueous bulk. With respect to the dilute aqueous phase (reference state), activity coefficients for the charged form of each conjugate pair are expected to increase more in the organic surface compared to the neutral species. This must be compensated for by a corresponding decrease in their relative abundances in the surface, leading to the apparent shift in p*K*_a_ as
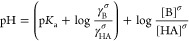
7When the charged species is the base, the shift is in the direction of higher p*K*_a_ and *vice versa* when it is the acid (Figure 4a).

In our experiments, surface shifts in  are broadly consistent with the relative differences in the surface enrichment of the individual acid and base conjugates obtained for binary aqueous solutions (Figure 3). For example, with p*K*_a_ = 4.9 for octanoic acid, a 0.1 M aqueous sodium octanoate bulk solution at neutral pH will comprise about 0.001 M octanoic acid, whereas we observe XPS peak ratios of  in the surface.^2^ From binary aqueous surface tension isotherms, the Monolayer model^14^ predicts individual *E*^σ^ values of 4990 and 371 for 0.001 M octanoic acid and 0.1 M sodium octanoate, respectively, corresponding to  for the surface.

The Langmuir adsorption model assumes an ideal dilute state with no interactions between adsorbing species in the surface. This is not expected when hydrophobic interactions significantly reduce the surface tension compared to that of pure water. Carboxylic acids and carboxylate anions form strong complexes,^64^ which could stabilize surface layers with roughly even proportions of the conjugates. At higher concentrations, species close to individual saturation may compete for surface adsorption. This is not seen in XPS spectra from our experiments, where concentrations were well below the reported solubility and/or CMC.^3^ Nonideal interactions could decouple adsorption into the mixed surface from binary solutions of the same concentrations. A simple relation between the solution composition and apparent shift in surface p*K*_a_ is therefore not expected.

### 3.3 Surface-Specific Chemistry

We investigated dilute aqueous mixtures of common atmospheric inorganic ions with surface-active carboxylate anion (C_*x*_^–^) salts at near-neutral pH.^1,2^ Specifically in mixtures with NH_4_^+^ ions, surface abundances of conjugate carboxylic acids (C_*x*_H) were further strongly enhanced, beyond what was observed in aqueous organic mixtures (Figure 4b). For a given bulk concentration, this did not involve a simultaneous decrease in the surface abundance of the carboxylate anions. Therefore, total abundances of the organic pair ([C_*x*_^–^] + [C_*x*_H]) in the surface were enhanced in NH_4_^+^ mixtures. This enhancement was stronger for the more surface-active pairs. Bulk-sensitive ^1^H NMR spectra confirmed the vast majority of the carboxylic acid−carboxylate anion pair to be on the ionic form, in complete agreement with the measured solution pH. The significant enhancement of C*_x_*H observed with XPS must therefore be specific to the solution surface.

The ammonium cation may act as a proton donor in aqueous solutions. Surface adsorption of C_*x*_^–^ and coadsorption of electrostatically attracted NH_4_^+^ cations form an ion-pair layer with strongly enhanced local abundances, increasing the probability of net proton transfer
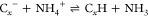
8according to Le Chatelier’s principle. The evaporation of ammonia from the surface may further contribute to irreversibly perturb the protonation equilibrium between NH_4_^+^ and C_*x*_^–^, leaving a surplus of carboxylic acid. We observed gas-phase NH_3_ evaporating from the solutions, as clearly distinguishable from solvated aqueous ammonia and ammonium in recorded N 1s XPS spectra. A strong surface enhancement of octanoic acid was also observed in aqueous mixtures of octanoate and diethanolammonium (DEA), the corresponding acid of diethanolamine (NH–(C_2_H_4_OH)_2_), which is much less volatile than ammonia (Henry’s law coefficients 3.87 × 10^–8^ atm L mol^−1^ and 1.61 × 10^–2^ atm L mol^−1^, respectively, at 25 °C).^2^ The octanoic acid fractions of the carboxylic C 1s signal were somewhat smaller for solutions with DEA (0.34) than with NH_4_^+^(0.46) but smallest (0.23) without either (Figure 4b). The evaporation of ammonia from the solution is therefore not necessary to drive the strong enhancement of the carboxylic acid form in the surface but likely amplifies the effect.

## 4 Atmospheric Significance

Liquid microjets have enabled surface-sensitive XPS experiments for aqueous solutions of surface-active organics as model systems for atmospheric aerosols and cloud droplets. We have gained direct, quantitative, molecular-level insights into the surface, which are not readily accessible with other methods. Even for these simple systems, we observed complex surface structures and chemistry, which are highly distinct from the solution bulk. These define the chemical and phase state, reactant species, and accessible functional groups governing rates, pathways, and products of heterogeneous and surface-specific reactions.

Surface adsorption can significantly deplete the bulk of submicrometer droplets, leading to increased water activity.^7,12,13,26,27^ The formation of bilayers between ionic surface-active species and coadsorbed counterions can further promote bulk depletion, decreasing water uptake but increasing the existing potential for aqueous solvation.^35^ The surface-specific (potentially irreversible) conversion of organic ions to their more surface-active neutral conjugates could significantly decrease the aqueous surface tension. This may promote atmospheric droplet growth by the condensation of water and other semivolatile species, thereby influencing cloud–climate interactions.

As the surface becomes increasingly saturated with adsorbing organic species, the more outward orientation of the alkyl chains presents a more hydrophobic surface to the gas phase. This may decrease droplet growth from the uptake of water and other hydrophilic species and shield hydrophilic groups from gas-phase reactions while making alkyl chains more available. The evaporation of small neutral molecules formed in surface-specific reactions, including NH_3_^1,2^ but potentially many others such as HCl^65^ from
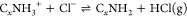
9could lead to significant mass loss from the aerosol phase. When the evaporating species have (Brønsted) acid−base character, it could also dramatically change the droplet acidity. Such a mechanism may contribute to the strong variation of observed pH for atmospheric aerosols.^41^

These surface-mediated mechanisms may profoundly affect the evolution of atmospheric chemistry due to the large volume fraction of aerosol and cloud droplets comprising their surfaces but are currently not taken into account in atmospheric models.^30,31,66^ Thermodynamic frameworks considering only the surface excess or a single adsorbing component are too simple to capture these variations of the droplet state.^13,26,27^ We found that the chemical environment of the surface changes in highly specific ways with concentration, pH, and cosolutes in the bulk. For aqueous droplets in the atmosphere, these may vary greatly with the ambient environment and air-mass history. By a somewhat fortuitous coincidence, the liquid microjet temperature during our measurements is immediately relevant for droplets in a rising air parcel or a cloud in the atmosphere.

### 4.1 Outlook

Faint and complex signals may prohibit applications of liquid microjet XPS to actual atmospheric samples (we tried). Immediate applications for atmospheric research involve the characterization of increasingly complex model systems and realistic experimental conditions. This will be crucial to validating and guiding new developments of models connecting surface and bulk composition−property relations.

**Figure 5:**
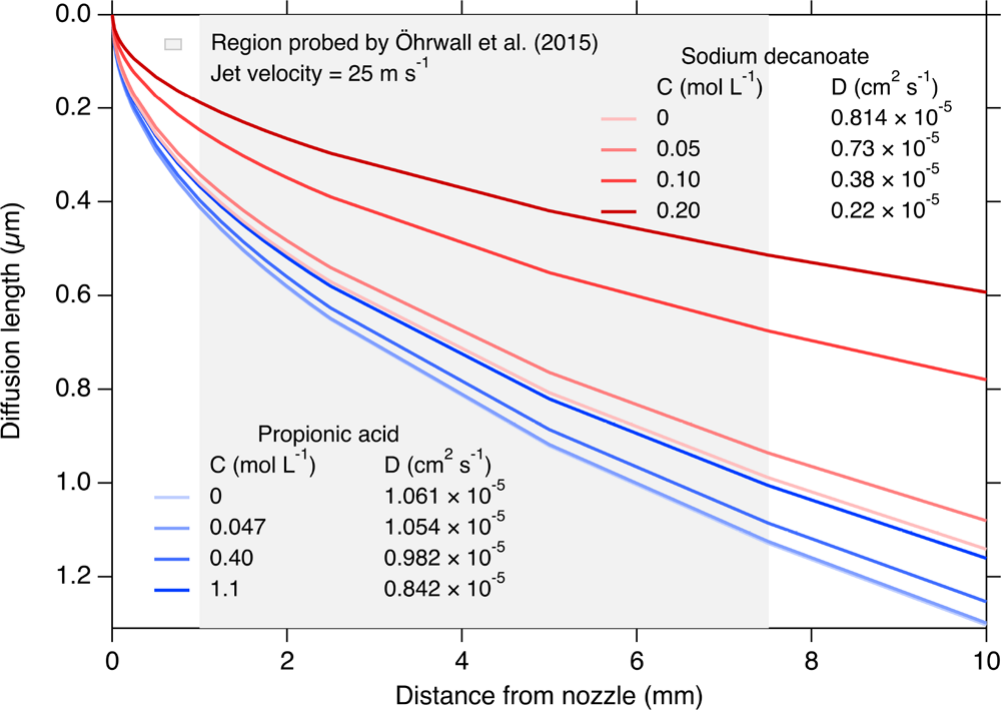
Estimated maximum diffusion distance for the surface adsorption of decanoate anions and propionic acid, indicating the depth into the microjet which has equilibrated with the surface at the time of measurement at different positions on the microjet. Diffusion coefficients (D) are from refs (68) and (69). Concentrations C = 0 mol L^−1^ indicate values extrapolated to infinite dilution.

Applications of liquid microjet XPS to more complex atmospheric model systems would benefit from several methodological developments. It is not known to what extent the surface of the high-speed liquid microjet has equilibrated at the time of measurement. In our experiments, we found no change in the phenomena observed by varying the intersection point of the X-ray beam with the jet,^2^ supporting similar previous assertions.^46^ The depth into solution with which the adsorption of surface-active solutes has equilibrated can be estimated from the solute diffusivity as 0.25–1.1 μm, 2 to 3 orders of magnitude greater than the surface thickness (Figure 5). Measurements at elevated, near-ambient pressures are possible with high-brilliance synchrotron lights sources, bringing the jet closer to steady state with respect to the gas phase and allowing the study of gas−solution heterogeneous reactions.^67^

The ability to extract absolute concentrations from liquid microjet XPS measurements without introducing overly simplifying assumptions will be pivotal for the quantitative analysis of surface-specific chemistry.^42,55^ In particular, absolute depth profiles for various solution components will reveal the extent of the nonisotropic surface region and constrain the impact of distinct surface properties. For example, surface depth profiles of conjugate acid−base pairs would resolve how much of the observed shifts can be attributed to different surface enrichments.^43^ Major challenges are the poor constraint on photoelectron EAL in different aqueous solutions and the limited number of depths possible to probe in each experiment. Advanced spectral analysis based on new machine learning methods has great potential for enabling this.^44,52^
